# Stress Resistance and Longevity Are Not Directly Linked to Levels of Enzymatic Antioxidants in the Ponerine Ant *Harpegnathos saltator*

**DOI:** 10.1371/journal.pone.0014601

**Published:** 2011-01-27

**Authors:** Sebastian A. Schneider, Charlotte Schrader, Anika E. Wagner, Christine Boesch-Saadatmandi, Juergen Liebig, Gerald Rimbach, Thomas Roeder

**Affiliations:** 1 Department Zoophysiology II, Christian-Albrechts-University of Kiel, Kiel, Germany; 2 Institute of Human Nutrition and Food Sciences, Christian-Albrechts-University of Kiel, Kiel, Germany; 3 School of Life Science and Center for Social Dynamics and Complexity, Arizona State University, Tempe, Arizona, United States of America; University of Liverpool, United Kingdom

## Abstract

**Background:**

The molecular mechanisms of variations in individual longevity are not well understood, even though longevity can be increased substantially by means of diverse experimental manipulations. One of the factors supposed to be involved in the increase of longevity is a higher stress resistance. To test this hypothesis in a natural system, eusocial insects such as bees or ants are ideally suited. In contrast to most other eusocial insects, ponerine ants show a peculiar life history that comprises the possibility to switch during adult life from a normal worker to a reproductive gamergate, therewith increasing their life expectancy significantly.

**Results:**

We show that increased resistance against major stressors, such as reactive oxygen species and infection accompanies the switch from a life-history trait with normal lifespan to one with a longer life expectancy. A short period of social isolation was sufficient to enhance stress resistance of workers from the ponerine ant species *Harpegnathos saltator* significantly. All ant groups with increased stress resistances (reproducing gamergates and socially isolated workers) have lower catalase activities and glutathione levels than normal workers. Therewith, these ants resemble the characteristics of the youngest ants in the colony.

**Conclusions:**

Social insects with their specific life history including a switch from normal workers to reproducing gamergates during adult life are well suited for ageing research. The regulation of stress resistance in gamergates seemed to be modified compared to foraging workers in an economic way. Interestingly, a switch towards more stress resistant animals can also be induced by a brief period of social isolation, which may already be associated with a shift to a reproductive trajectory. In *Harpegnathos saltator*, stress resistances are differently and potentially more economically regulated in reproductive individuals, highlighting the significance of reproduction for an increase in longevity in social insects. As already shown for other organisms with a long lifespan, this trait is not directly coupled to higher levels of enzymatic and non-enzymatic antioxidants.

## Introduction

Animal species differ vastly in how long they live. Some species have life expectancies of only a few days, whereas others can live for to be several hundred years old. To unravel the mechanisms underlying the evolution of long lifespan, a large number of natural and genetically tractable systems have been employed in ageing research [1]. The major aim of these studies has been to understand how nature controls ageing. One major common result of these studies was that prolonged lifespan appears to be associated with increased stress resistance [2], [3], [4].

The sole intervention that consistently has been shown to increase longevity throughout the animal kingdom is caloric or dietary restriction (CR or DR). Although CR mediated lifespan extension has been the topic of numerous studies, the underlying molecular mechanisms are poorly understood [5], [6]. Apparently, both, cell-autonomous and non-cell-autonomous effects contribute to CR mediated effects. Several studies have demonstrated that systems involved in nutrient level sensing are central for CR mediated lifespan prolongation and thus being relevant for ageing related processes in general. Consequently, a number of different pathways have been shown to be involved in the phenomenon, comprising the target of rapamycin (TOR), the AMP-activated protein kinase, those converging onto sirtuin activation and the insulin signalling pathway [7], [8]. Proteins with a central integrator function within these pathways are e.g. SMK-1, PGC1alpha and FoxO factors [9], [10]. Activation of these systems is believed to increase stress resistance and life expectancy by changing the transcriptional profile of the cells. Among the target genes are those coding for enzymatic antioxidants and chaperones. This would be in line [5], [7] one of the earliest molecular theories of ageing, which proposed that reactive oxygen species (ROS) progressively damage macromolecules, leading to a decline in cellular function and, finally, to death [11]–[12]. Increased longevity induced by ectopic expression of enzymatic antioxidants in nematodes, flies, and mice supports the hypothesis that reducing oxidative damage is important for prolonging lifespan [4], [13], [14], [15].

Although, analysing long-lived mutants of genetically tractable model organisms supplied us with a wealth of information regarding those genes that are involved in lifespan determination, some drawbacks of this approach have to be acknowledged. Work with *Drosophila* revealed that fly strains differ dramatically in their lifespan and their responsiveness to longevity interventions such as CR [16]. Thus, complementary studies using systems based on more natural population structures would greatly increase the value of these results. One approach compared the molecular and cellular mechanisms by which long-lived species differ from short-lived species have been employed. Small rodents can vary in their life expectancy by more than an order of magnitude. Under optimal laboratory conditions, mice can reach an age of 4 years, whereas naked mole-rats (*Heterocephalus glaber*), which are of similar size, can live up to 30 years. Surprisingly, these extremely long-lived small rodents exhibit no evidence of enhanced antioxidant defence compared with mice [17]. Although naked mole-rats show significantly higher levels of markers for oxidative damage compared to mice, no ageing associated increase in these parameters could be observed, which contrasts the situation found in mice [18]. One major problem associated with these comparative studies is the genetic disparities between short- and long-lived species that may account for at least some of the differences between them. Therefore, genetically similar or even identical model organisms with disparate life expectancies should be much better suited for studying ageing. The only group of organisms that readily meet this criterion are eusocial insects [19], [20]. Eusocial insect colonies are characterized by the occurrence of phenotypically different but genetically very similar castes, which usually not only fulfil different tasks within the group but which also have different life expectancies. In bees, queens live up to ten times longer than workers [21]. Some ant species show an even more impressive divergence in their life expectancies, as individual queens of the garden ant *Lasius niger* can reach ages of more than 28 years, whereas workers usually die after 1-2 years [22], [23]. Surprisingly, increased longevity is neither correlated with levels of antioxidant enzymes [22] nor with telomere lengths in the garden ant [24]. In eusocial insects, the long-living individuals are usually the reproductive ones, which stands in contrast to observations that reproduction and longevity are inversely correlated [1]. The mechanisms underlying this increased longevity in reproductive social insects are still not understood, but it has been hypothesized that production of vitellogenin, which has antioxidant properties, may be responsible [25], [26].

**Figure 1:**
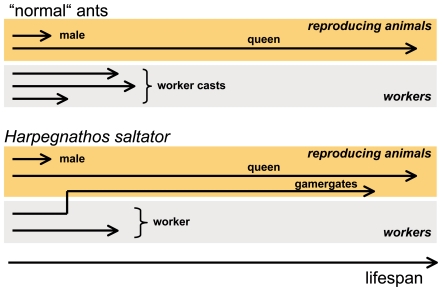
Differential life expectancies in ant colonies. Members of a “normal” ant colony have widely varying life expectancies. Reproductive animals (males and queens) differ substantially in their lifespan compared to non-reproductive individuals. The non-reproductive animals, corresponding to members of different worker castes, have lower life expectancies (top) than the queen. In the ancestral ponerine ant *Harpegnathos saltator*, fully developed workers have the ability to transform into reproductive individuals, called gamergates, which increases their life expectancy several fold (bottom).

Queens and workers usually differ in their ovarian development (and thus the ability to produce offspring), but also in various other traits such as individual morphology, physiology and nutrition, which makes assignment of increased lifespan to one of these traits almost impossible. Very few eusocial hymenopterans possess a type of social organisation, which enables adult workers to become reproductive individuals, or gamergates. This transition from worker to gamergate is accompanied by a more than doubling of life expectancy, which has been shown for species of at least two different ponerine genera [27], [28]. Thus, these species enable us to study the mechanisms underlying increased lifespan, while excluding other confounding influences. *Harpegnathos saltator*, the model we choose for our studies, can endure as a colony with secondary polygyny after the initial stage as a queenright colony (Fig. 1). It has very recently been introduced as a model organism for the study of eusocial insects [29]. In older colonies, a group of inseminated, egg-laying workers supersede the late queen [30]. Workers that make the transition to dominant gamergates, increase their lifespan more than twofold. Taking advantage of this unique system, we tested a number of hypotheses central to ageing research: 1) longevity is correlated with a general increase in stress resistance, 2) antioxidant systems are of central importance for the extension of lifespan, and 3) the switch from normal to extended lifespan can be induced experimentally.

## Results

*Harpegnathos* colonies tend to be very long lived in the laboratory and can be maintained for much more than a decade. In the course of our experiments, we have never observed workers older than 1.5 years, whereas we observed several gamergates with lifespans of much more than 3 years [31]. These characteristics are congruent with those observed in other ponerine ant species [27], [28].

### Differential susceptibility of workers and gamergates to major stressors

**Figure 2:**
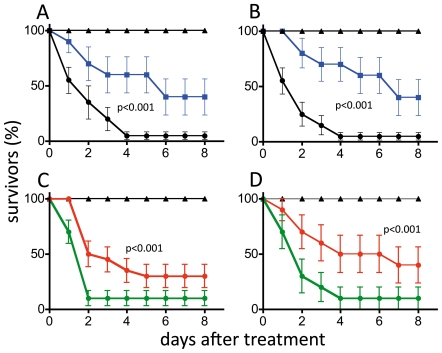
Survival rates of treated ants. Survival curves for workers (solid circles) and gamergates (solid squares) injected with live *Erwinia carotovora* (A, C) or with paraquat (B, D). The percentage of survivors is plotted against the time following injection (± S.E.M.). The control animals (triangles) were injected with the same volume of sterile LB-medium instead of bacterial suspension. Statistical analysis was performed with Cox regression analysis; p values are given in the figure. Survival curves for gamergates (solid squares - blue) and workers (solid circles - black) that were injected with the *Erwinia carotovora* (A) or the ROS generating compound paraquat (B). Workers isolated for 24 h prior to the experimental injections (solid circles - red), showed a statistically significant increased survival following injection of pathogens (matching controls; solid circles - green) (C) and paraquat (D). The number of animals per experimental group was n = 30. Mean values are given ± S.E.M., p-values are listed in the figures.

Workers and gamergates responded differently to the experimentally induced infection (Figure 2A) or injection of paraquat (Figure 2B). Following infection, gamergates had a significantly higher survival rate than workers (n = 30 animals per group, Cox regression analysis, chi^2^ = 8.6, hazard ration 2.86, p<0.001). Gamergates also show a statistically significant higher survival rate than workers following injection of paraquat (statistics as above, chi^2^ = 13.3, Hazard ratio 4.0, p<0.001). Even after eight days, 40% of the gamergates survived, whereas almost all infected workers (more than 95%) died within the first 4 days following experimental infection (Figure 2A). The experimental procedure itself (tested by injection of similar amounts of sterile LB medium) had no influence on survival of either gamergates or workers.

### Effects of social isolation on stress resistance

Workers isolated 24 h before infection showed a significantly higher survival rate than the workers that were isolated following treatment (n = 30 animals per group, Cox regression analysis, chi^2^ = 13.63, Hazard ration 2.3, p<0.001). Nevertheless, the susceptibility of both groups of workers was still higher than that of gamergates (Fig. 2C). Injection of paraquat showed similar effects as the bacterial infection when pre-isolated workers and workers were compared (statistics see above, chi^2^ = 11.1, Hazard ratio 2.9, p<0.001) (Fig. 2D). The individually housed animals were more resistant to paraquat injection than the socially housed individuals. Remarkably, isolation after injection had a negative effect as workers in this group were more susceptible to paraquat injection than either of the other two experimental groups, and the survival rates were also lower than those of treated normal workers (Fig. 2C, D). We found no behavioural peculiarities in the corresponding ants. A brief dissection following the experiments revealed that the one-day isolation period did not yet lead to a visible led to the development of ovaries.

### Role of ROS-detoxifying enzymes in longevity

**Figure 3:**
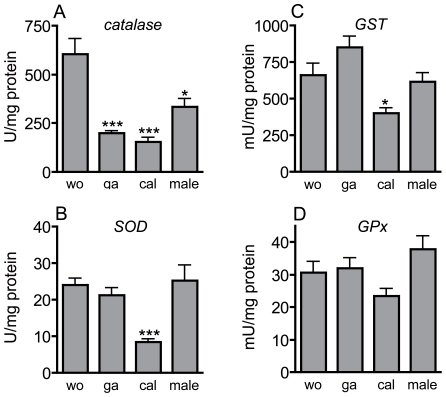
Enzyme activities in different groups of ants. Four naturally occurring groups of adult *Harpegnathos* were subjected to a detailed analysis regarding their antioxidant enzyme activities. Individual workers were used for analysis and the corresponding enzyme activities as well as the protein concentrations were measured and the enzyme activities per mg of protein plotted. Usually, 10 independent measurements were made for every category. The groups under investigation were workers (wo), gamergates (ga), callows (cal), and males (male). Workers and gamergates were identified by means of an in depth analysis of their social behaviour. Statistically significant differences are marked by asterisks (* p<0.05, ** p<0.01, *** p<0.001). Catalase (A) statistical analysis with Dunnett-T-test after ln-transformation, SOD (B), glutathione-S-transferase activity (GST) (C), and glutathione peroxidase activities (GPx) were measured (D). Statistical analysis for SOD, GST and GPx was performed with the Games-Howell-test.

Workers had the highest catalase activity, followed by males, which showed a significantly reduced activity (n = 10 for all experimental groups, one-way ANOVA, Dunnett t-test after ln-transformation, t = 3.1, p<0.05) (Fig. 3A). The two other groups, gamergates and callows, showed significantly reduced levels of catalase activity, with only approximately 30% of the activity measured in the other groups (n = 10 gamergates and callows compared with workers, statistics as above, t = 5.1 and 6.33 respectively, p<0.001 each)(Figure 3A). SOD activities were similar in all groups of ants, with the exception of the callows, which had approximately 30% of the SOD activity measured in the other groups. The difference between workers and callows was statistically significant (n = 10 for all experimental groups, on-way ANOVA, Games-Howell test, t = 7.78, p<0.001) (Fig. 3B). The two major enzymes of glutathione metabolism exhibited a different trend. GST activity was slightly higher in gamergates than in workers, but this difference was not statistically significant (gamergates 801±195 mU/mg; workers 660±250 mU/mg). Males had GST activity almost identical to that of workers and only the callows had significantly reduced activity if compared with that of workers (n = 10 for all experimental groups, one-way ANOVA, Games-Howell test, t = 3.24, p<0.05) (Fig. 3C). Glutathione peroxidase activities were statistically indistinguishable between all groups of ants (n = 10 for all experimental groups, Dunnett-T-test after ln-transformation, p>0.1 each) (Fig. 3D).

**Figure 4:**
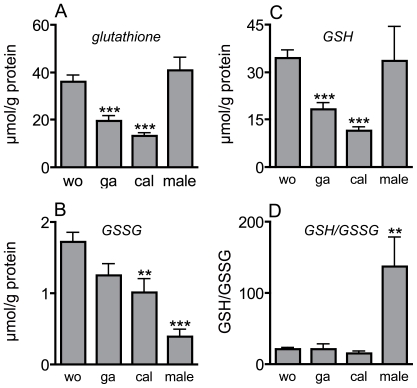
Glutathione metabolism in different groups of ants. Different parameters of the glutathione metabolism were quantified. The experimental setup was exactly as described in figure 3. The glutathione level (A), the levels of oxidised glutathione (GSSG) (B), those of reduced glutathione (GSH)(C), as well as the ratios of reduced and oxidised glutathione (D) were measured. Statistically significant differences are marked by asterisks (* p<0.05, ** p<0.01, *** p<0.001). Number of independent samples for workers and gamergates were n = 10, for callows n = 7 and for males n = 4. Analyses in A and C were performed with the Games-Howell-test, in B using the Dunnett-T-test after ln-transformation, in D using the Mann-Whitney-U-test.

A detailed analysis of glutathione metabolism itself revealed differences that partially mirror those observed for catalase activity. The glutathione content of workers and males was almost identical and was significantly higher than that of gamergates and callows (n = 10 for workers and gamergates, n = 7 for callows and n = 4 for males, one-way ANOVA, Games-Howell test, t = 5.02 and 6.96, p<0.001 for workers *vs* gamergates and for workers *vs* callows). Gamergates had approximately 50% of the glutathione content observed in workers and callows approximately 30% (Fig. 4A). Levels of oxidized glutathione (GSSG) were also much lower in gamergates (not significantly) and callows (n = 10 for workers and gamergates, n = 7 for callows and n = 4 for males, one-way ANOVA, Dunnett t-test, t = 3.97, p<0.05) compared to workers (Fig. 4B). Males had an even lower concentration, which was also statistically significant (statistics as above, t = 7,8, p<0.001). Similarly, levels of reduced glutathione were lower in gamergates and callows than workers (n = 10 for workers and gamergates, n = 7 for callows and n = 4 for males, one-way ANOVA, Games-Howell-test, t = 5.13 and 8.04, p<0.001 for workers *vs* gamergates and for workers *vs* callows) (Fig. 4C). Consequently, the ratios between reduced and oxidized glutathione were almost identical in workers, gamergates, and callows. In contrast, males had an approximately ten fold higher ratio than workers (n = 10 for workers and gamergates, n = 7 for callows and n = 4 for males, one-way ANOVA, Mann-Whitney U-Test, t = −4.68, p<0.01) (Fig. 4D).

**Figure 5:**
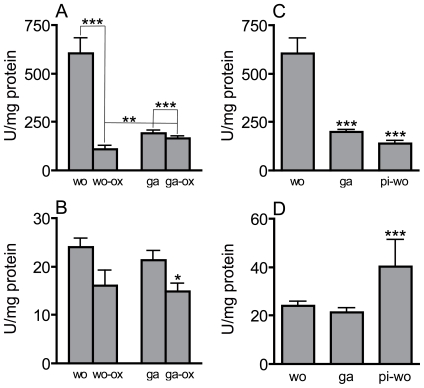
Effect of oxidative stress and pre-isolation on catalase and SOD activities. Catalase (A, C) and SOD (B, D) activities of experimentally manipulated animals were measured. The experimental setup was exactly as described in figure 3. Workers (wo) and gamergates (ga) were subjected to oxidative stress (wo-ox and ga-ox) or workers were pre-isolated 24 h before measurement (pi-wo). Effect of paraquat injection on catalase activities (A) and SOD activities (B), as well as pre-isolation on catalase activities (C) and SOD activities (D) were determined. Statistically significant differences are marked by asterisks (* p<0.05, ** p<0.01, *** p<0.001). Analyses in A and C were performed with the Dunnett-T-test after ln-transformation, in B and D using the Games-Howell-test.

We also measured catalase and SOD activities under high oxidative stress conditions induced by injection of a paraquat solution into the hemocoel. Injection of paraquat into the hemocoel of workers resulted in a dramatic reduction of catalase activity (n = 10 for all experimental groups, one-way ANOVA, Dunnett t-test after ln-transformation, t = 9.07, p<0.001). In gamergates, which have a much lower basal catalase activity, the reduction was only slight but also highly significant (statistics, see above, t = 8.37, p<0.001). The catalase activity of stressed gamergates was significantly higher than that of stressed workers (statistics, see above, t = 4.83, p<0.01 treated workers *vs* treated gamergates) (Fig. 5A). For SOD activities, similar patterns were observed in workers and gamergates. ROS-injection induced a slight decline in the SOD activity in both experimental groups. This decline was found to be significant in gamergates only (n = 10 for all experimental groups, Games-Howell test, t = 3.84, p<0.05) (Fig. 5B). Pre-isolation completely changed the enzymatic behaviour of workers. The catalase activities were significantly lower than those of workers (n = 10 for both groups, Dunnett-T-test after ln-transformation, t = 9.12 p<0.001) (Fig. 5C) and the SOD activity significantly higher (n = 10 for all experimental groups, Games-Howell test, t = 8.05, p<0.001)(Fig. 5D).

## Discussion

One of the long-standing dogmas of ageing research has been that stress resistance and longevity are positively correlated [3], [32], [33]. Here, we show that in a natural system, the switch from a caste with normal life expectancy to one with a much longer life expectancy is indeed associated with significantly higher stress resistance. This increased resistance can be observed for different stressors such as ROS and pathogen contact. *Harpegnathos*, like some other ponerine ants, has the peculiar ability to switch from a non-reproductive to a reproductive lifestyle during adult life, thus excluding any confounding differences that may originate developmentally. Recent in-depth analyses of long-lived mutants have identified non-genetically based reasons for increased longevity [34], thus further supporting the usefulness of the natural systems such as *Harpegnathos* for ageing research. As already pointed out, major parameters relevant for ageing research are strictly strain dependent in model organisms, highlighting the role of the genetic background. The colonies used in our study had been obtained in the 90ies from related sampling sites as gamergate-right colonies and maintained since than as inbred colonies, thus representing a situation comparable to that of a certain fly strain or isofemale line. Therefore, using animals from 10 different colonies is comparable to an experimental approach where 10 different isofemale lines are taken, a diversity that should cover most of the genetic variability observable in a natural population [35].

Surprisingly, the age of colonies in the wild does not correlate with the observation in the lab, because relatively short colony survival times have been reported. This discrepancy may be accounted to extrinsic factors such s heavy monsoon rains, drought or predators [36], but regarding the usefulness of *H. saltator* as a model in ageing research, its quantifiable characteristics in lab cultures are much more relevant.

### Social isolation increases stress resistance

Of special interest is the observation that a short period of social isolation has a significant protective effect. In isolated workers, stress resistance against ROS reached levels similar to those observed in gamergates, whereas resistance against infections was intermediate between that observed in workers and gamergates, indicating that different mechanisms are involved. The 24 h period of social isolation is not sufficient to induce visible signs of ovary maturation. In addition, animals were supplied with sufficient nutrients to exclude starvation as a confounding variable. Mild starvation, known as dietary restriction, is the most consistent way to increase lifespan in almost all animals studied [5], [37]. Some ants appear to be extraordinarily starvation resistant as colonies of the myrmecine ants *Temnothorax rugatulus* can survive several months of complete starvation [38]. Thus, the effects of social isolation cannot be attributed to starvation, which would also induce an increased stress resistance via mechanisms similar to those operative in dietary restriction. *Harpegnathos* workers have the ability to found a colony on their own at least under laboratory conditions [39]. Under normal conditions, workers mate with males produced in the colony and the sperm is stored for long periods. Isolation of these inseminated but infertile workers taken from a normal colony is sufficient to transform them into reproducing animals that lay eggs and produce offspring, which may lead to the foundation of a new colony [39].

Our results imply that as soon as inhibitory fertility signals from dominant females are removed, the process of transformation to a reproductively active individual is initiated. Even before the ability to produce offspring (maturation of ovaries) is detectable, the increased stress resistance occurs. Therefore, we can speculate that removal of the inhibitory factors from the fertile animals in the colony triggers a multifaceted response, which includes ovarian development on the one hand, but which, on the other hand, also alters other factors governing stress resistance.

### Role of enzymatic detoxification of ROS in stress resistance and survival

Since Harman introduced his free radical theory of ageing [11], antioxidants, either enzymatic or non-enzymatic, have been considered of central importance in lifespan control [2], [4], [13]. This common ground in ageing research has recently been destabilized, generating doubts as to the simple correlation between increased enzyme levels and increased lifespan [40], [41]. Those animals that are normally long-lived tend to have lower amounts of antioxidant enzymes such as catalase or SOD [18], [42], an observation that has also been made in social insects [22], [43]. A more complex correlation between oxidative stress and longevity emerges if recent results regarding the life prolonging effects of mild stressors are taken into account. Hormesis, chronic mild stress, has a significant lifespan enhancing effect [44], [45], [46]. Recently, it has been shown that downregulation rather than upregulation of catalase activity increases lifespan in the yeast *Saccharomyces*, presumably also via induction of hormesis [47]. Reactive oxidant species are not only metabolic by-products that induce ageing, they are also signalling molecules within the organism. This dual function of oxidant/antioxidant systems should nevertheless not compromise the interpretation of antioxidant activities measured in whole animals, because the signalling function is usually spatially restricted and accounts only for a small percentage of the overall activity.

In the different groups of *Harpegnathos* individuals, we have a similarly complex pattern of anti-oxidant activities. We measured catalase and SOD as the most relevant ROS detoxifying enzymes, but also measured enzymes involved in glutathione metabolism to obtain a largely complete picture. Surprisingly, gamergates, the long-lived reproductive individuals, have significantly lower catalase activity than workers. This mirrors the situation observed in honey bee queens [43]. Another group of ants, the callows, corresponding to the youngest individuals in the colony, have a similarly low catalase activity, further indicating that stress resistance or age and levels of enzymatic antioxidants are not positively correlated. The similarities between the enzymatic characteristics of gamergates and callows hold true also for the total, as well as the reduced, glutathione contents. Although gamergates tend to be older (or, at least, are assumed to be older) than normal workers, they show enzymatic characteristics closely resembling those observed in the youngest animals. The interpretation of the low levels of antioxidant enzymes in the youngest animals is more complex, as they require strong oxidative activities to enable darkening of their cuticle when they make their conversion to “normal” workers. However, the observation that the ratio between reduced and oxidized glutathione is similar in callows, workers and gamergates argues against this possibility. The very high values of glutathione and the high ratio of GSH/GSSG in males may be interpreted as prearrangements to cope with the highly energy demanding maiden flight.

A possible reason for this counterintuitive regulation of antioxidant activities was provided by another experiment. Stressing animals by injection of the ROS-generating compound paraquat induced a dramatic drop in the catalase activity of workers, whereas the drop in activity in gamergates was only marginal. A comparable situation has been observed in foraging honey bees. Young foraging bees increase their catalase activity approximately 3 fold following prolonged flight. Old foragers experience a dramatic drop in catalase activity following this procedure [48]. This supports the assumption that workers have to cope with high oxidative stress during their normal life and that their reserve pool for this enzyme is not sufficient to cope with additional external stress. It appears that the long-lived individuals may experience lower levels of oxidative stress, allowing them to survive with less ROS-related enzymatic activity than the short-lived individuals. This difference may allow them to regulate expression of the enzymes in times of stress over a greater range, which should be beneficial for longevity.

Ponerine ants such as those of the genus *Harpegnathos* offer the unique opportunity to study the transition towards increased lifespan as part of the normal life history trait. When workers shift to reproduction as a gamergate, they also change the regulation of stress resistance, which may be causally linked to increased lifespan. Epigenetic mechanisms have recently been proposed to be candidates mediating these long lasting switches in life history traits. In addition, insulin-signalling appears to be modulated, which may lead to changes in typical target genes of the transcription factor Foxo, namely enzymatic antioxidants [29]. Even though gamergates show an increased resistance to major stressors such as infection or oxidative stress, they seem to invest less in stress resistance than normal workers. In fact, their very low levels of catalase activities and glutathione levels resemble more the pattern of young individuals, which suggests that the life confined to inside the nest allows for the lower investments in stress resistance. A switch towards more stress resistant animals can also be induced by a brief period of social isolation that may already be associated with a shift to a reproductive trajectory. In *Harpegnathos saltator*, different systems of stress resistance are differently and potentially more economically regulated in reproductive individuals, highlighting again the significance of reproduction for an increase in longevity in social insects.

## Materials and Methods

### Colony maintenance and experimental groups

Individual ants for all experimental procedures were taken from a total of 17 gamergate-right colonies. These colonies were collected as gamergate-right colonies in the years 1994, 1995 and 1999 in the area of Jog Falls in Karnataka State, South India and kept under laboratory conditions as inbred colonies since then. The colonies were housed in plastic boxes (19 cm×19 cm×10 cm) containing a floor of plaster with a carved out nest chamber covered with a glass plate. The colonies were provided with live crickets *ad libitum* and housed at 25°C under a 12 h/12 h light/dark regime. Ants were taken from fully reproductive colonies that fulfilled the following criteria: i) the minimum size of the colony was 80 ants, ii) the colonies contained eggs, larvae and pupae, iii) the colonies showed an established and stable dominance hierarchy.

### Sampling strategy

To exclude colony dependent biases, we used a matching sample strategy. At the same time, this strategy ensures that a population covering genetic diversity is reflected in the experiments. For survival experiments, we took a total of 20 animals from 10 different colonies. Two gamergates and two workers each were removed from the respective colonies at the same time and used for the experiments, meaning that genetic material from 10 different colonies is represented in each experiment. For the social isolation experiments, an identical sampling strategy was used (2 workers for pre-isolation, 2 workers for post-isolation from 10 different colonies each). Regarding the enzymatic determination experiments, a similar sampling strategy with lower numbers of animals was used (equal numbers – usually two each- of gamergates, workers and callows from a peculiar colony). Only sampling of males didn't follow this stringent sampling strategy due to the temporal restriction in male availability. Thus, usually 2 males were taken from one colony for one type of experiments.

### The experimental groups were categorized as follows

Gamergates. Gamergates were initially identified by the dominance behaviour they showed towards their nestmates. Their status was checked by a quick dissection prior to homogenisation (in the case of the enzyme assays) or after death or at the end of the experiments (in the case of the survival assays). Even though we did not track the gamergates for more than a few weeks, we can estimate their minimum age. It takes about two to three month that a colony becomes socially stable again after the establishment of new gamergates (JL pers. observ.) In addition, the very young workers below four weeks of age do not become gamergates in the presence of slightly older workers. Given that gamergates can live more than three years [31], it is save to assume that average gamergate age is much more than six months. Workers. Workers were collected after they had been monitored for at least 15 min to make sure that they performed a task inside as well as outside the nest. This procedure excluded any bias that could occur through an age dependent shift in behaviour as found in honey bees. Again, their status was checked by a quick dissection prior to homogenisation (in the case of the enzyme assays) or after death or at the end of the experiments (in the case of the survival assays). Since workers rarely exceed one year of age and the number of older workers is naturally smaller than that of younger workers, it is unlikely that the average worker age is above six months. Callows. Callows were identified by the light brown colour of their cuticule, which contrasts to the dark brown colour of older adults. Since the ants fully lose this colour about 30 days after hatching from the pupae, all animals in this group were younger than one month. Males. Males were collected directly from the inside of the nest. Older males that had already left the nest area were excluded from the experiments.

Additionally, a group of socially isolated workers was created as follows. Workers were collected from the colonies, as described above, and transferred to plastic tubes (diameter 6 cm, height 12 cm) where each individual was kept isolated. The tubes had a plaster floor with an embedded 1.5 ml Eppendorf tube (brown plastic) as a shelter. During isolation, the ants were provided with fresh crickets every day and kept under the same environmental conditions as the colonies. In addition, ingestion of food was monitored regularly to exclude confounding effects caused by starvation. A control group of workers was kept isolated following the injection procedure only. A short overview of the different experimental groups Is given in table 1.

**Table 1:** Experimental groups of ants – characteristics and treatment

group	reproductive status	age range	treatment
gamergate	fully reproductive	1 month–4 years	colony-injection-colony
worker	not reproductive	1 month–1.5 years	colony-injection-colony
callow	not reproductive	0–30 days	colony-injection-colony
isolated worker	not reproductive	1 month–1.5 years	colony-injection-isolation
preisolated worker	not reproductive	1 month–1.5 years	isolation-injection-isolation

Listed are all groups of ants that were used in this study. In addition, their reproductive state, their anticipated age range, as well as the timing of the experimental treatment are listed. Colony means that the animals were in the colony until they were treated. Isolation means a short period (before treatment 24 h) of single animal housing was used.

Workers and gamergates may differ not only in their reproductive state, but also in their mean age (gamergates may usually be older than workers). To exclude these potential age related, confounding variables between workers and gamergates, we performed another set of experiments, where we randomly grouped workers into three experimental categories, thus excluding any age-related bias. In addition, social isolation is known to be able to induce fertility in workers and the ability to found a new colony [39]. The individuals in the first group were held separately in individual cages and fed *ad libitum* with crickets for 1 day, and then injected with either bacteria or paraquat and held individually from that point on. Animals from the first control group were taken from the same colonies and used for injection of either bacteria or paraquat as described above, and, thus, never experienced individual housing. A second group of controls was also taken from the colonies, injected, and then housed individually following the experimental manipulation.

### Survival assays - Injection procedure

Workers and gamergates were taken directly from the colony whereas isolated workers were housed individually for 24 h. The animals were immobilized by keeping them on ice for a few minutes. The ants were then injected with 40 nl of medium (either containing the stressor or pure medium as a control) into the gaster and marked with enamel paint. After allowing them to recover for a few minutes, the ants were either reinserted into their colonies or into their isolation tubes. For the social isolation experiment, workers were kept isolated for 24 h before the induction of experimental stress. Matching controls were treated directly after separation from the colony. After treatment with the stressor, the animals were kept in their housing tubes for 8 days and the survivors were counted every 24 h. Ants were dissected and checked for the developmental status of their ovaries either after death or at the end of the survival assay. We did not observe aversive behaviours towards injected animals until they were dead, thus premature death caused by aversive behaviours of nestmates can be excluded.

Ants were infected by injecting them with 40 nl of LB medium containing *Erwinia carotovora* at an OD of 3.6 or with 40 nl of sterile LB medium. Colonies and isolation tubes were checked for dead animals every 24 h for 8 days. Because the ant mortality assay contained censored data (i.e., some ants were still alive at the end of the experiment), we applied a Cox regression analysis (SAS version 9.0: Proc PHREG) to test for the effect of ‘life-history stage’ on survival of the ants.

Ants were injected with 40 nl of Schneider's *Drosophila* medium containing 500 mM paraquat as the experimental treatment or 40 nl of sterile Schneider's only. The paraquat solution was prepared immediately prior to the injection procedure and used within 15 min. Observation procedures and statistical analysis were the same as those described above for the infection experiments.

### Quantification of enzyme activities

To elucidate whether enzymatic antioxidants are responsible for the differing stress resistances and life expectancies observed in the *Harpegnathos saltator* groups, we quantified the activities of systems involved in ROS detoxification. Two groups of enzymes are central to ROS detoxification: 1) catalase and superoxide-dismutase (SOD), and 2) enzymes involved in glutathione metabolism, namely glutathione-S-transferases (GST) and glutathione peroxidases (GPx). Levels of reduced (GSH) and oxidized glutathione (GSSG) were measured as well. Workers and gamergates were the major experimental groups used in these assays, but very young workers, callows, as well as males and experimentally manipulated workers and gamergates were examined also.

The ants were collected as described above. The ants that were exposed to paraquat were collected from the colonies 24 h after the injection procedure and the isolated ants were housed individually for 24 h prior to these procedures. Individual ants were used as single samples. Ants were killed by freezing at −80°C. Subsequently, each ant was homogenized in liquid nitrogen, resuspended in 110 µl of cold 1x PBS and vortexed. To remove debris, samples were centrifuged at maximum speed in a tabletop centrifuge at 4°C for 5 min. The samples were then frozen at −80°C in aliquots of 50 µl until further use. Determination of enzymatic activity was usually performed with 10 samples handled independently. For some determinations slightly lower numbers were used (glutathione in males, n = 4; in callows, n = 7; catalase following induction of oxidative stress, n = 9; SOD following oxidative stress, n = 7).

The enzyme assays were carried out as follows. Superoxide-Dismutase. SOD Activity was determined according to the methods of Marklund and Marklund [49], which is based on the inhibition of autoxidation of pyrogallol by SOD. Catalase. The method of Johansson and Borg [50], which measures the production of formaldehyde from a hydrogen donor (methanol) with the chromogen purpald, was used to measure catalase. Glutathione-S-Transferase. The GST activity measurements were conducted as described previously [51]. In this assay, the rate of glutathione conjugation to the substrate CDNB (1-chloro-2,4-dinitrobenzene) is measured. Glutathione-peroxidase activity. GPx activity was measured according to the method of Lawrence and Burk [28], which utilizes a coupled enzymatic assay, beginning with the reduction of cumene hydroperoxide. Glutathione-content. The total glutathione content, as well as the oxidized and reduced glutathione, were quantified according to well established methods [52], [53].

### Statistical analyses

The statistical evaluation of the survival assay data (infection with *Erwinia carotovora* and injection of paraquat solution) was carried out by using the Cox regression analysis (SAS version 9.0: Proc PHREG). The number of animals per experimental group was n = 30. The data concerning the level of glutathione and the activities of the various enzymes were treated as following: The arithmetical means and the standard errors of each group were calculated. Further statistical evaluation was carried out using SPSS Version 13.0. To test whether a statistical difference to the control group exists, a one-way ANOVA (analysis of variance) and a Levene-test for homoscedasticity was used. If homoscedasticity was found, the Dunnett-test was applied. If homoscedasticity was not on hand, the test after Games-Howell was carried out. Gaussian distribution was check with Kolmogrorw-Smirnow and Shapiro-Wilk. In case of non-Gaussian distribution, the data were ln-transformed and the non-parametric Mann-Whitney-U-Test was applied. Numbers of independent samples are listed in the figure legends.
